# The Effect of Statins on Serum Vitamin D Concentrations Among Older Adults

**DOI:** 10.7759/cureus.8950

**Published:** 2020-07-01

**Authors:** Carlos H Orces, Martha Montalvan, Daniel Tettamanti

**Affiliations:** 1 Rheumatology, Laredo Medical Center, Laredo, USA; 2 Investigación Biomedicina, Universidad De Granada España, Granada, ESP; 3 Investigacion, Universidad Espíritu Santo, Guayaquil, ECU; 4 Nutricion Clinica, Universidad Catolica De Santiago De Guayaquil, Guayaquil, ECU; 5 Medicina, Universidad De Granada, Granada, ESP; 6 Internal Medicine, Hospital Luis Vernaza, Guayaquil, ECU; 7 Medicina Interna, Universidad Católica De Santiago De Guayaquil, Guayaquil, ECU

**Keywords:** statins, vitamin d levels, older adults

## Abstract

Background

Randomized and observational studies have previously reported inconsistent results for the direct association between statin therapy and 25, hydroxyvitamin D [25(OH)D] levels. Thus, the present study aimed to examine the relationship between statin use and 25(OH)D and its metabolites concentrations in a large nationally representative sample of older adults.

Methods

This study was conducted using data from the National Health and Nutrition Examination Survey. Participants were asked to show the medication containers of all the products used in the previous 30 days, and the prescription of statins was defined on the three-level nested therapeutic classification scheme of Cerner Multum’s Lexicon. General linear models adjusted for potential confounders were created to compare 25(OH)D concentrations between older adults taking statins and those who did not.

Results

A total of 6,261 participants with a mean age of 69.5 years comprised the study sample. Of those, 40.2% were taking statins with a median length of therapy of 5 years. Adjusted mean 25(OH)D_3_ and 25(OH)D levels were 3.3 and 4.4 nmol/L higher among participants taking statins than those who did not, respectively. Moreover, this association was consistently seen regardless of the duration of therapy and particularly in subjects taking simvastatin, atorvastatin, or rosuvastatin. In subgroup analyses according to BMI categories and vitamin D intake, higher 25(OH)D levels were also seen among statin users. By contrast, this association was attenuated among those with a daily vitamin D between 400 and 800 and >800 IU.

Conclusion

Older adults on statin therapy had significantly higher serum 25(OH)D concentrations. Additional research should be conducted to define the mechanism of this association and determine if the pleiotropic effects attributed to statins may be mediated by vitamin D.

## Introduction

3-hydroxy-3-methylglutaryl-coenzyme A reductase inhibitors (statins) have been widely used as a lipid-lowering therapy in the primary and secondary prevention of atherosclerotic cardiovascular disease [1-3]. A recent analysis of the National Health and Nutrition Examination Survey (NHANES) described that prescription of cholesterol-lowering drugs increased with age from 17.4% in adults aged 40-59 to 47.6% in adults aged 75 years and older during 2011-2012. Moreover, among adults prescribed cholesterol-lowering medication, 83% used a statin alone [4].

Although the precise mechanism of statin therapy on 25(OH)D metabolism is not fully clarified, researchers have postulated that since 7-dehydrocholesterol is the common precursor of vitamin D and cholesterol, the inhibition of 3-hydroxy-3-methylglutaryl-coenzyme A reductase may increase 7-dehydrocholesterol levels providing an adequate substrate for the synthesis of 25-hydroxyvitamin D [25(OH)D] [5]. The occupation of the active site of the CYP3A4 enzyme by statins may also account for increased serum 25(OH)D levels [6]. Previously, few observational studies and randomized controlled trials reported conflicting results regarding the effect of statins on 25(OH)D concentrations. Some studies described higher 25(OH)D concentrations among statin users, whereas others did not [7-15]. Nevertheless, these studies were limited by small sample size, subjects with acute coronary syndrome, individual generic statins, diabetics hospitalized, and postmenopausal women with osteopenia [7,9,11,14-15]. Therefore, the purpose of this study was to examine the relationship between statin use and 25(OH)D concentrations in a nationally representative sample of older adults.

## Materials and methods

The NHANES is a biannual cross-sectional study conducted by the National Center for Health Statistics of the Centers for Disease Control and Prevention. The purpose of the NHANES is to collect data about the health, nutritional status, and health behaviors of the noninstitutionalized civilian resident population of the U.S. Information about the analysis and reporting guidelines of the NHANES are described elsewhere [16].

Characteristics of participants

The demographics file provides individual, family, and household level information on the following topics: The six-month time period when the examination was performed, demographic information about the household reference person, and other selected demographic information, such as gender, age, race/Hispanic origin, and education. The Department of Health and Human Services guidelines were used as the poverty measure to calculate the ratio of family income to poverty. 

In the Mobile Examination Center, BMI was calculated as body weight in kilograms divided by height in meters squared and then rounded to one decimal place. Questionnaires on alcohol consumption and cigarette smoking were also administered. As previously reported, the physical activity questionnaire is based on the Global Physical Activity Questionnaire and includes questions related to leisure-time activities. The reported number of days and time (in minutes) spent performing vigorous or moderate leisure-time physical activity in the previous week were calculated, and according to the 2008 Physical Activity Guidelines for Americans, participants were categorized as physically active, insufficiently active, or physically inactive [17]. Moreover, participants were asked about their general health, which was categorized as good to excellent and fair to poor. The diagnosis of diabetes was established if participants reported a physician diagnosis of diabetes or had an HbA_1c_ ≥6.5% [18]. Cardiovascular disease was defined based on an affirmative response to any one of four questions about doctor-diagnosed heart attack, angina, coronary heart disease, or stroke [4]. Similarly, participants were considered to have high cholesterol if they affirmatively responded to the question “Have you ever been told by a doctor or other health professional that your blood cholesterol level was high?”

Vitamin D intake

Since the NHANES cycle 2007-2008, vitamin D intake has been collected from the types and amounts of foods and beverages consumed during the 24-hour period prior to the interview. All NHANES participants responding to the dietary recall interview were also eligible for the dietary supplement questions. The participant’s use of vitamin D supplements was reported as a mean vitamin D dosage over the course of 30 days prior to the household interview. Data were routinely examined for discrepancies and erroneous entries [19].

25(OH)D and its Metabolite Concentrations

The CDC developed the standardized liquid chromatography-tandem mass spectrometry (LC-MS/MS) to measure 25(OH)D_3_, 25(OH)D_2_, and total 25(OH)D levels. For the LC-MS/MS method, total 25(OH)D (in SI units of nmol/L) was defined as the sum of 25(OH)D_3_ and 25(OH)D_2_. However, due to rounding off, the sum of 25(OH)D_3_ and 25(OH)D_2_ will not necessarily be equal to the 25(OH)D. This method has better analytical specificity and sensitivity compared to immunoassay methods and fixed analytical goals for imprecision (≤10%) and bias (≤5%) [20].

Statin prescription

During the household interview, participants were asked if they have taken medications in the past 30 days for which they needed a prescription. Those who answer “yes” were asked to show the interviewer the medication containers of all the products used. For each drug reported, the interviewer recorded the product’s complete name from the container. Prescription medications were classified based on the three-level nested therapeutic classification scheme of Cerner Multum’s Lexicon. Cholesterol-lowering medications were identified using the second level of drug category code 19, and the third level of drug category code 173 was used to identify statins [4]. The following drugs were included in the present analysis: lovastatin, pravastatin, simvastatin, fluvastatin, atorvastatin, cerivastatin, rosuvastatin, and pitavastatin.

Statistical analysis

The characteristics of the study population were stratified according to statin therapy and compared using the chi-squared test and the t-test for categorical and continuous variables, respectively. General linear models adjusted for the six-month study period, age, gender, race/ethnicity, education, income, BMI, smoking status, alcohol use, physical activity, self-reported health status, diabetes, and vitamin D intake were created to compare 25(OH)D_2_, 25(OH)D_3_, and total 25(OH)D concentrations between statin users with their non-user counterparts. Similarly, the independent associations between generic statins, the duration of statin therapy (< 2, 3-6, and >6 years) and 25(OH)D concentrations were explored. Since a small number of participants (n = 5) were prescribed fluvastatin, cerivastatin, or pitavastatin, these drugs were excluded from this analysis. Moreover, in subgroup analyses, the modifying effects of BMI categories and vitamin D intake (< 400, 400-800, and >800 IU) on 25(OH)D levels were examined according to statin therapy status. Statistical analyses were performed using SPSS Complex Sample software, V.17 (SPSS Inc, Chicago, Illinois, USA) to incorporate constructed weights for the combined survey cycles and obtain unbiased, national estimates representative of the older U.S. population. A P-value <0.05 was considered statistically significant. Of 7,859 participants aged 60 years and older in the NHANES cycles 2007-2008 through 2013-2014, those with missing data on 25(OH)D concentrations (n = 990), BMI (n = 514), and dietary vitamin D (n = 945) were excluded from the analysis. In general, participants with missing data were more likely to be examined during the winter months, female, had less than high school education, physically inactive, and self-reported fair to poor health.

## Results

A total of 6,261 participants with a mean age of 69.5 years comprised the sample size. Of these, 40.2% reported used statins and the median duration of therapy was 5 years. Table 1 shows the characteristics of participants stratified according to statin therapy. Overall, statin therapy was higher in men, non-Hispanic whites, high-school graduates, former smokers, and those with fair to poor health compared with those who did not. Moreover, as expected, statins were more frequently prescribed among diabetics and those with cardiovascular disease. In general, mean 25(OH)D_2_ and total 25(OH)D concentrations were significantly higher among statin users than their non-user counterparts. 

**Table 1:** Characteristics of the participants according to statin therapy RIP, Ratio of family income to poverty; AA, Associates of Arts Parenthesis represents standard error of the estimates.

	Statin users (n = 2,456)	Non-users (n = 3,805)	P value
Six-month period, %			0.816
Nov 1^st^ to Apr 30^th^	38.7 (3.4)	38.3 (3.4)	
May 1^st^ to Oct 31^th ^	61.3 (3.4)	61.7 (3.4)	
Age (years), mean	70.4 (0.2)	69.0 (0.1)	<0.0001
Gender, %			<0.0001
Male	51.0 (1.1)	42.0 (0.8)	
Female	49.0 (1.1)	58.0 (0.8)	
Race/ethnicity, %			<0.05
Hispanics	6.2 (0.9)	7.8 (1.0)	
Non-Hispanic white	81.1 (1.3)	80.3 (1.5)	
Non-Hispanic black	7.7 (0.8)	8.0 (0.8)	
Others	5.0 (0.6)	3.9 (0.4)	
BMI (kg/m^2^), mean	29.8 (0.1)	28.4 (0.1)	<0.0001
Education, %			<0.05
Less than high school	21.8 (1.5)	19.4 (1.2)	
High school graduate	25.7 (1.4)	22.4 (0.9)	
Some college or AA degree	28.4 (1.4)	29.1 (1.0)	
College graduate or above	24.1 (1.4)	29.1 (1.6)	
RIP, %			0.101
<1.00	8.6 (0.7)	9.7 (0.6)	
≥1.00	91.4 (0.7)	90.3 (0.6)	
Smoking status, %			0.0001
Never	45.2 (1.5)	51.7 (1.1)	
Former	44.5 (1.4)	37.5 (0.9)	
Current	10.8 (0.7)	10.8 (0.7)	
Alcohol use, %			0.470
Yes	70.1 (1.4)	69.0 (1.4)	
No	29.9 (1.4)	31.0 (1.4)	
Physical activity status, %			0.342
Inactive	56.3 (1.6)	56.6 (1.4)	
<150 min/week	14.7 (0.9)	16.2 (0.8)	
≥150 min/week	29.0 (1.4)	27.2 (1.3)	
General health condition, %			<0.05
Good to excellent	75.6 (1.2)	80.2 (0.9)	
Fair to poor	24.4 (1.2)	19.8 (0.9)	
Diabetes, %			<0.0001
Yes	33.2 (1.2)	15.9 (0.9)	
No	66.8 (1.2)	84.1 (0.9)	
High cholesterol, %			<0.0001
Yes	82.2 (0.9)	38.9 (1.1)	
No	17.8 (0.9)	61.1 (1.1)	
Cardiovascular disease, %			<0.0001
Yes	66.0 (1.8)	34.0 (1.8)	
No	33.7 (1.0)	66.3 (1.0)	
Vitamin D intake (IU), %			<0.05
<400	43.5 (1.4)	48.5 (0.9)	
400-800	22.1 (0.9)	21.2 (1.1)	
>800	34.4 (1.2)	30.3 (1.1)	
25(OH)D (nmol/L), mean			
Total 25(OH)D	79.0 (1.0)	75.4 (0.8)	<0.05
25(OH)D_3_	71.5 (1.0)	69.8 (0.9)	0.081
25(OH)D_2_	7.5 (0.7)	5.6 (0.4)	<0.05

As shown in Table 2, after adjustment for potential confounders, 25(OH)D_3_ and 25(OH)D levels were on average 3.1 and 4.4 nmol/L higher among older adults taking statins than those who did not, respectively. In contrast, a non-significant 1.4 nmol/L difference in 25(OH)D_2_ levels was seen among older adults on statin therapy. 

**Table 2:** The association between statin therapy and 25(OH)D levels and its metabolites among older adults Model 1 adjusted for study period, age, gender, race/ethnicity, education, income, BMI, smoking status, alcohol use, and physical activity. Model 2 adjusted for model 1 and self-reported health status, diabetes, and vitamin D intake.

Vitamin D concentrations	Statin users (SE)	Non-users (SE)	Mean difference (SE)	P-value
25(OH)D_3_ (nmol/L)				
Mean	71.5 (1.0)	69.8 (0.9)	1.7 (0.9)	0.081
Model 1	72.9 (0.9)	69.1 (0.8)	3.8 (0.9)	<0.0001
Model 2	72.4 (0.8)	69.3 (0.7)	3.1 (0.8)	<0.05
25(OH)D_2 _(nmol/L)				
Mean	7.5 (0.7)	5.6 (0.4)	1.9 (0.6)	<0.05
Model 1	7.5 (0.7)	5.7 (0.4)	1.8 (0.6)	<0.05
Model 2	7.2 (0.7)	5.8 (0.4)	1.4 (0.6)	0.054
Total 25(OH)D (nmol/L)				
Mean	79.0 (1.0)	75.4 (0.8)	3.6 (1.0)	<0.05
Model 1	80.5 (1.0)	74.8 (0.7)	5.7 (0.9)	<0.0001
Model 2	79.6 (0.8)	75.2 (0.7)	4.4 (1.0)	<0.0001

**Figure 1:**
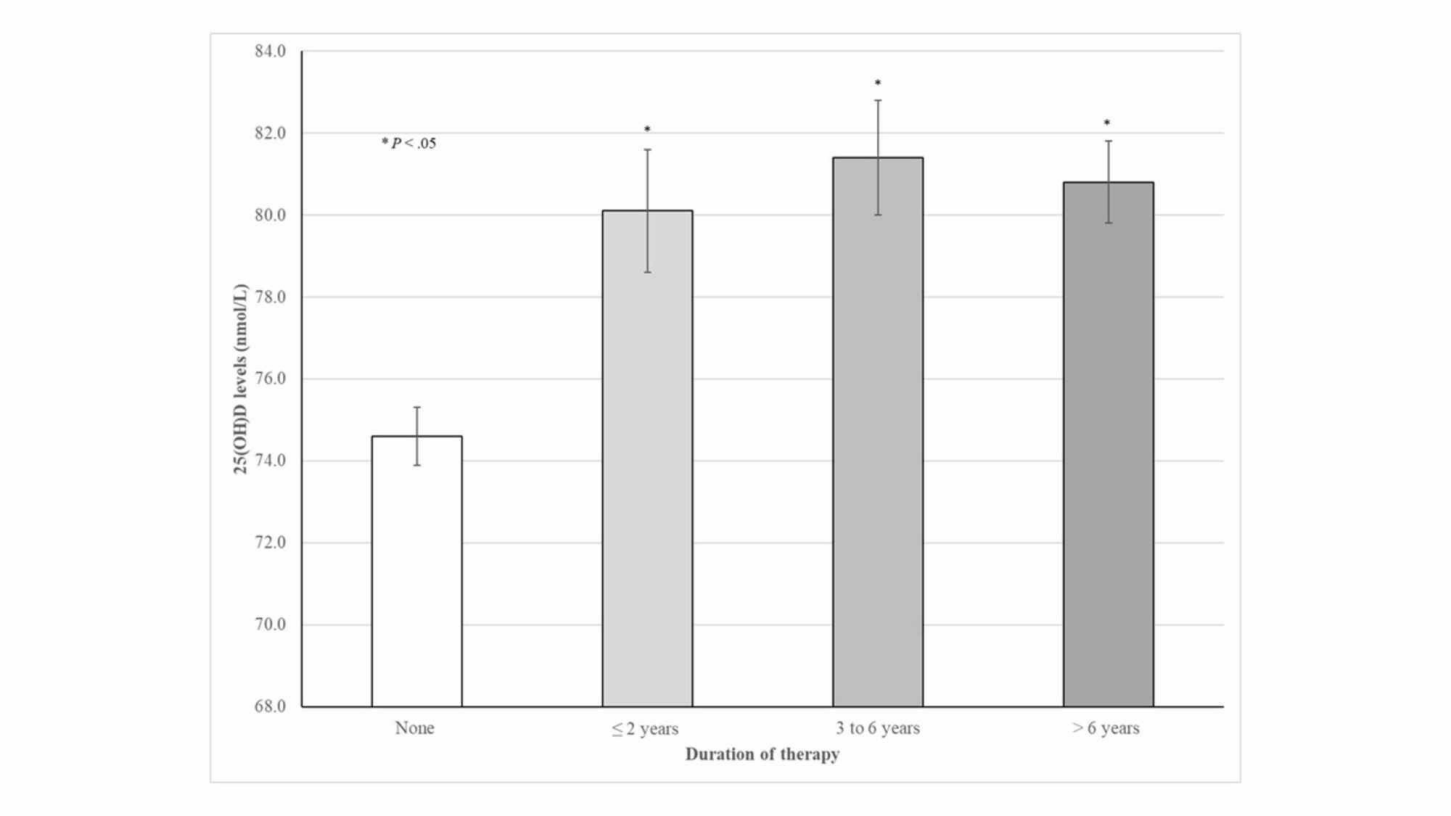
25(OH)D concentrations according to duration of statin therapy among older adults

As shown in Figure 1, adjusted mean 25(OH)D concentrations were consistently higher among older adults taking statins irrespective of the duration of treatment. For instance, those taking statins >6 years had on average 6.2 nmol/L higher 25(OH)D concentrations than those who did not. 

**Figure 2:**
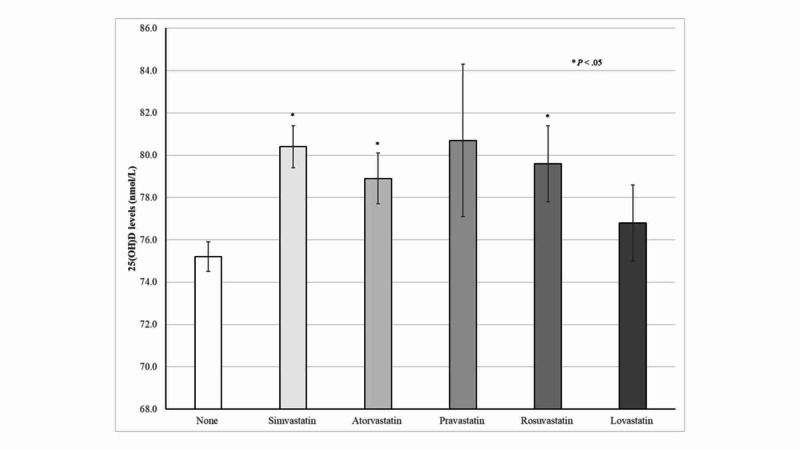
25(OH)D concentrations according to generic statin therapy among older adults

As shown in Figure 2, overall higher 25(OH)D concentrations were consistently seen among participants on any generic statin therapy. However, these associations were statically significant among subjects taking simvastatin, atorvastatin, or rosuvastatin. 

**Figure 3:**
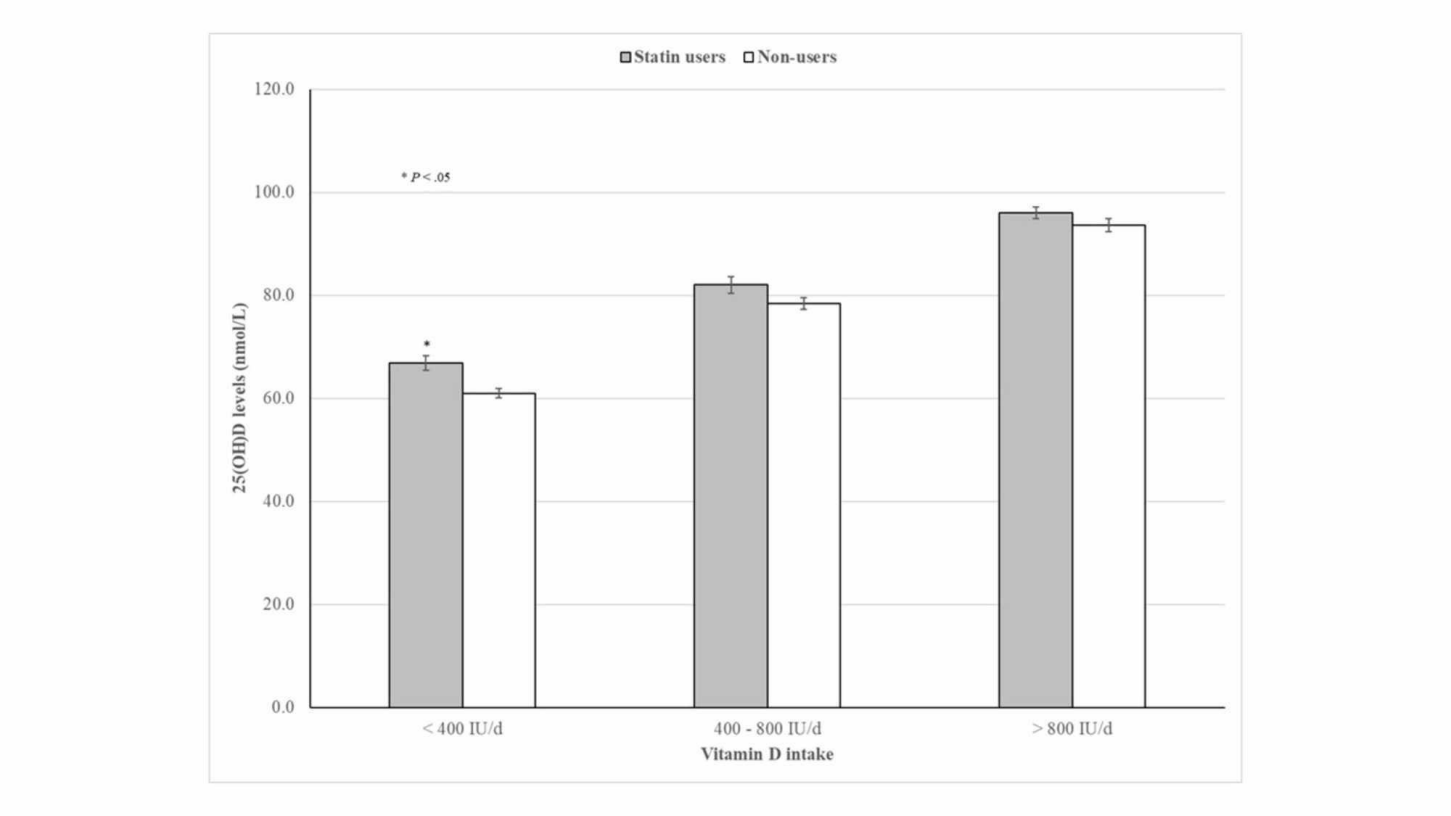
25(OH)D concentrations according to statin therapy and vitamin D intake

As shown in Figure 3, 25(OH)D concentrations progressively increased with an increase in vitamin D intake. However, no significant differences in 25(OH)D levels were seen between statin users compared with their non-user counterparts, particularly among participants taking vitamin D between 400 and 800 IU and >800 IU per day. 

**Figure 4:**
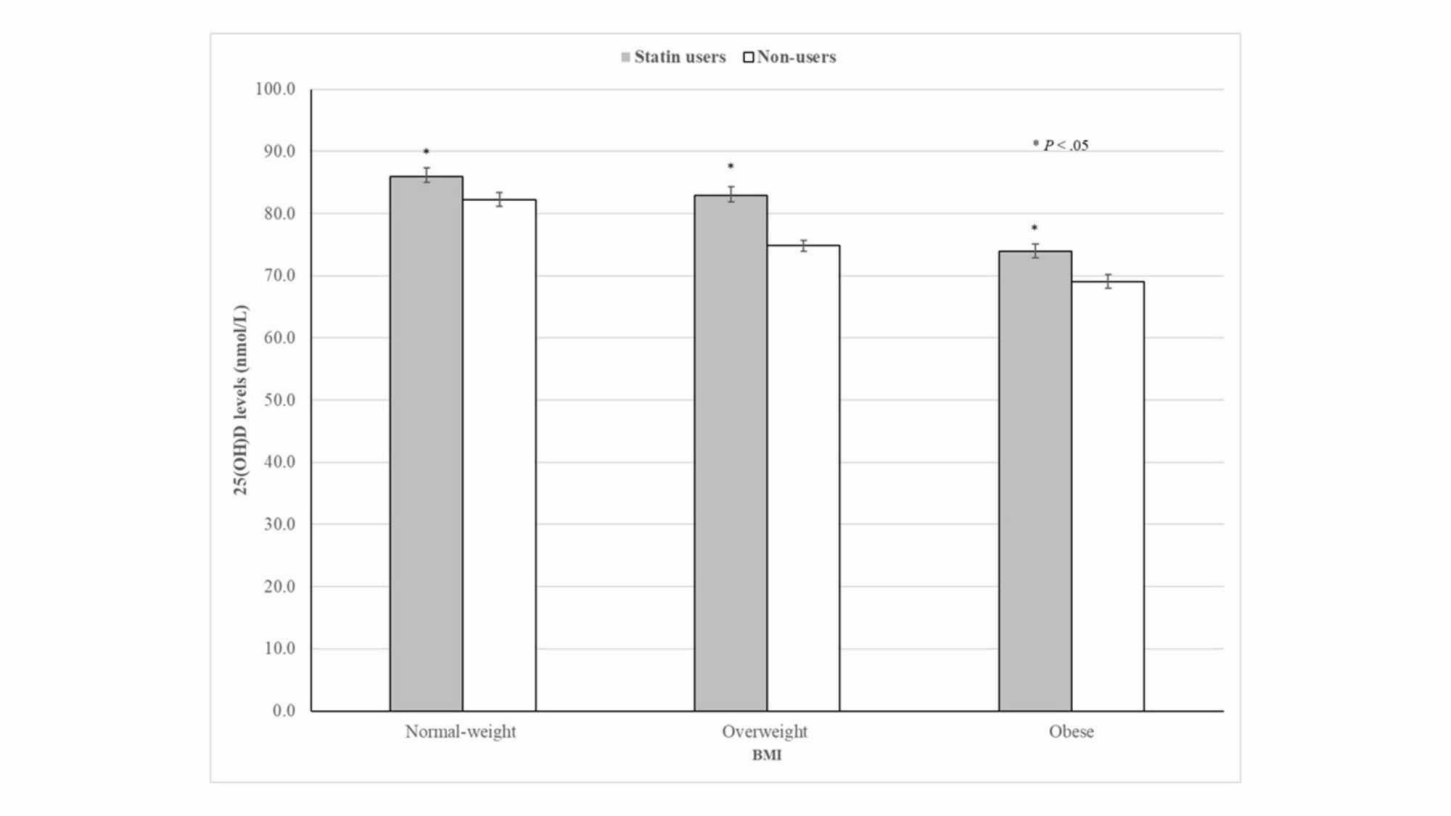
25(OH)D concentrations according to statin therapy and BMI categories BMI, body mass index

As expected, 25(OH)D concentrations were inversely correlated with BMI categories. Despite this evidence, higher 25(OH)D levels were seen among normal-weight, overweight, and obese participants on statin therapy than those who did not (Figure 4). 

## Discussion

The present findings indicate that serum 25(OH)_3_ and total 25(OH)D concentrations were significantly higher among statin users than their non-user counterparts. Notably, these associations were consistently seen after adjustment for potential confounders, irrespective of the duration of therapy, or generic statin prescribed. Our findings contrast with those from a recent systematic review and meta-analysis conducted by Mazidi *et al*., in which inconclusive effects of statins on 25(OH)D concentrations were reported. Indeed, the pooled estimate (weighted mean difference) of the effect of statin therapy on serum vitamin D from randomized clinical trials was 2.71 ng/ml and decreased by -0.70 ng/ml in observational studies. These contradictory findings were attributed to small sample sizes of the included studies and different doses and duration of vitamin D supplementation [5].

In subgroup analyses, we found that among participants with dietary vitamin D intakes between 400 and 800 IU or >800 IU per day, 25(OH)D concentrations did not significantly differ between participants on statin therapy and those who did not. However, increased 25(OH)D levels were seen among older adults on statin therapy and a daily intake of <400 IU per day. Since dietary vitamin D intake from food and supplements is the main determinant of vitamin D status in older adults, increased 25(OH)D levels among statin users were particularly seen among those with inadequate vitamin D intake [21]. In addition, Bischoff-Ferrari *et al*. in a pooled analysis of three double-blind randomized clinical trials suggested that lower 25(OH)D response to vitamin D supplementation may be a class effect of all statins, which differ according to vitamin D dosing regimens [13]. 

Notably, higher 25(OH)D concentrations were consistently seen among statin users, irrespective of the duration of therapy. Moreover, mean 25(OH)D concentrations minimally differed between statin therapy <2 years compared with their counterparts with >6 years. Thus, the present findings suggest that statins have a sustained effect on 25(OH)D levels, which was seen even at the beginning of therapy. Similarly, higher 25(OH)D concentrations were seen among participants according to individual generic statins use. This association was statistically significant among participants taking simvastatin, atorvastatin, or rosuvastatin. Indeed, subjects taking simvastatin and atorvastatin, the most frequently prescribed statins, had on average 5.2 and 3.7 nmol/L higher 25(OH)D levels compared with non-users, respectively. In contrast, Renjmark *et al*. in a small randomized controlled trial among healthy postmenopausal women reported no significant differences in plasma 25(OH)D levels between subjects treated with simvastatin 40 mg/day for a year compared with their counterparts receiving placebo [15]. Likewise, Mazidi *et al*. previously demonstrated in a randomized, double-blind, placebo-controlled trial lasting 30 days that simvastatin 40 mg/day did not have a significant effect on serum 25(OH)D levels [7]. Possible explanations for these contradictory results were attributed to unmeasured predictors of vitamin D status such as vitamin D intake and sun exposure [15]. Lower 25(OH)D concentrations have been consistently reported across different latitudes in obese subjects compared with their normal-weight counterparts, which have been related to limited sun exposure, decreased bioavailability of vitamin D in fat tissue, and lower vitamin D supplements use [22-25]. As expected, the present results also demonstrated an inverse relationship between BMI and 25(OH)D concentrations among older adults. Despite this evidence, normal-weight, overweight, and obese participants taking statins had significantly higher 25(OH)D concentrations than those who did not. 

Of relevance, the beneficial effects of statin therapy on coronary artery disease have been described as not only aggressive reduction of circulating pro-atherogenic lipid particles but also to its pleiotropic effects, including improvement of endothelial function and anti-inflammatory and anti-oxidant actions, whose mechanism is presently undetermined [26]. Moreover, it has been postulated that the pleiotropic effects of statins may be mediated by vitamin D [9]. In fact, in agreement with this hypothesis, previous studies have demonstrated that lower 25(OH)D concentrations have been inversely associated with the progression of aortic calcification and myocardial infarction [27-28]. 

The present study has several limitations that should be mentioned. First, because of the NHANES cross-sectional design, these results do not necessarily infer causation. Second, the statin dosage was not available in the prescription medication section. Thus, the intensity of statin therapy on 25(OH)D concentrations may not be established. Third, sun exposure and sun-protective behavior characteristics, which may have a direct effect on the synthesis of 25(OH)D_3_, were not explored in the present analysis. Fourth, most sociodemographic and health variables were self-reported, which may have led to recall bias. Despite these limitations, this study clearly demonstrated that statin therapy is associated with increased 25(OH)D concentrations in a large nationally representative sample of older adults.

## Conclusions

Older adults on statin therapy had significantly higher 25(OH)D concentrations than those who did not. Further research is needed to precisely define the mechanism of this association and determine whether the pleiotropic effects attributed to statins may be mediated by vitamin D.
